# Transcultural adaptation and validation of the patient empowerment in long-term conditions questionnaire

**DOI:** 10.1186/s12913-017-2271-7

**Published:** 2017-05-04

**Authors:** Paloma Garcimartin, Josep Comin-Colet, Pilar Delgado-Hito, Neus Badosa-Marcé, Anna Linas-Alonso

**Affiliations:** 1 Cardiology Department, Hospital del Mar, Parc de Salut Mar, Barcelona, Spain; 2 Heart Diseases Biomedical Research Group, IMIM (Hospital del Mar Medical Research Institute), Barcelona, Spain; 3 Escuela Superior de Enfermería del Mar, Parc de Salut Mar, Barcelona, Spain; 4 Chronic Heart Failure Section, Cardiology Department and Community Heart Failure Unit, University Hospital Bellvitge. Hospitalet de Llobregat, Barcelona, Spain; 5 Cardiovascular Diseases Research Group, Research Programme in Inflammatory, Chronic and Degenerative Diseases, IDIBELL (Bellvitge Biomedical Research Institute), Hospitalet de Llobregat, Barcelona, Spain; 6 School of Nursing, University of Barcelona, Barcelona, Spain; 7 Bellvitge Biomedical Research Institute (IDIBELL), Hospitalet de Llobregat, Barcelona, Spain

**Keywords:** Patient participation, Self-efficacy, Decision making, Personal autonomy, Quality of life, Chronic heart failure, Metric properties, Questionnaires, Patient-reported outcomes, Nursing

## Abstract

**Background:**

Patient empowerment is a key element to improve the results in health, increase satisfaction amongst users and obtain higher treatment compliance. The main objective of this study is to validate the Spanish version of the questionnaire “Patient empowerment in long-term conditions” which evaluates the patients’ level of empowerment of chronic diseases. The secondary objective is to identify factors which predict basal empowerment and changes (improvement or deterioration) in patients with Heart Failure (HF).

**Methods:**

An observational and prospective design of psychometric type to validate a questionnaire (aim 1) and a prospective study of cohorts (aim 2). The study will include 121 patients with confirmed diagnosis of HF. Three measurements (basal, at 15 days and at 3 months) will be carried out: quality of life, self-care and empowerment. Descriptive and inferential analyses will be used. For the first aim of the study (validation), the test-retest reproducibility will be assessed through intraclass correlation coefficient; internal consistency will be assessed through Cronbach’s alpha coefficient; construct validity through Pearson’s correlation coefficient; and sensibility to change through effect size coefficient.

**Discussion:**

Set a valid questionnaire to measure the level of empowerment of patients with chronic diseases could be an effective tool to assess the results from the provision of the health care services. It will also allow us to identify at an early stage, those groups of patients with a low level of empowerment. Hence, they could become a risk group due to poor management of the disease, with a high rate of decompensation and a higher use rate of the health system resources.

## Background

Ageing population is the most important phenomenon of the last decades, with serious repercussions at a social and economic level. In 2009, 11% of the population was over the age of 60, it is calculated that this will increase to 22% of the population in 2050. The population over the age of 80 will see a higher growth rate [1]. The ageing in the population implies a greater prevalence in chronic diseases. In turn, the increase in chronic diseases, comorbidity and functional dependency will generate a high cost not only to the health system but also at a social, economic, and human level. Since, it will hinder individuals and communities to develop all their potential [2].

Every country has made an effort to design and implement strategies to manage more efficiently care to patients with chronic diseases with the objective of improving their quality of life and reducing the rate of health complications. Therefore, the health system has had to change its focus, centred on the most acute patients and cures, and adopt a more interdisciplinary vision; in which the system is patient centred. A model whose objective is to empower patients and enable self-management in the processes of the disease [3, 4].

The concept of empowerment is used in a great range of contexts. One of the first contexts we have reference of its use was found in Paulo Freire’s liberating education philosophy [5], he uses this concept as an answer to oppression and social inequality. Later the term was analyzed in community physiology, critic social theory, gender studies, rural economy studies, and finally, in health education and promotion [6–8].

In the health field, the term in its use, has also got a long history. Firstly, it was adopted as the key element to promote health, later it was used as a way to increase autonomy and the participation of the patients in decision making related to their health. Finally, with the increase of chronic pathologies, it is the strategy used so patients participate and take responsibility of their own care with the aim of improving their results in health and secondly, to control health costs [9–11].

Although a spread consensus over the importance of the term and its multidimensional character, there is not a unique definition for the concept or for the different dimensions it takes into account:

- Angelmar and Berman [10] use the proposed definition by the World Health Organization (WHO) for patient empowerment: a process through which people gain greater control over decisions and actions affecting their health. These authors specify that the necessary conditions for patient empowerment are: patients need to comprehend their new role (be more active, responsible and participative); improve their knowledge on their health and the different treatment options; develop abilities to carry out self-care action in a competent manner, to improve self-efficiency and; create a propitious environment so professionals can facilitate two levels of the process: motivation and health literacy.
- Varekamp [12] define it as a process to help patients develop knowledge, abilities and gain conscience of their needs. Simultaneously, it allows them to define their objectives, take responsibility of their treatment and increase their autonomy.
- Small [13] consider that empowerment is both a process and a result derived from the communication between professionals and patients. Through which, information is exchanged on the resources for the disease which increases self-control, self-efficiency, the abilities to confront the disease and the capability of achieving a change in their condition.
- According to Anderson [14] empowerment is also a process and a result. The aim in the process is to increase the capability of critical thinking and to act autonomously, whilst the result shows self-efficiency.

From this analysis we understand that the interventions of the professionals must be aimed to both the process and the result. With regards to the process, therapeutical relationships must be established to encourage continuity in the relationship, centred on the patient, to encourage decision making, incorporating abilities of confrontation and positive attitude [15–17]. In this regard, many authors consider that the main strategies to improve the process of empowerment are: active listening strategy and motivational interviews [18–20]. The nurses, due to their competencial features, are the most prepared to promote and facilitate support to self-care and accompany the patient in their process of empowerment [21–23].

Small [24] carried out a systematic revision of empowerment tool and assessed nine in relation to the following criteria: definition of empowerment, process of development of the scale, format, dimensions, content of the items and punctuation and psychometric properties of the tool.

The analysed instruments are:

- Patient Enablement Instrument (PEI) [25]
- Empowerment Scale (ES) [26, 27]
- Psychological Empowerment Questionnaire (PEQ) [28]
- Diabetes Empowerment Scale (DES) [14, 29]
- Patient Empowerment Scale (PES1) [30]
- Empirical Empowerment Measure (EMP) [31]
- Patient Empowerment Scale (PES2) [9]
- Client Empowerment Scale (CES) [32]
- Pregnancy Related Empowerment Scale (PRES) [33]

From this analysis carried out, we notice that only three of the instruments (PEI, ES and DES) present positive psychometric properties. Furthermore, they have all been developed in well defined contexts and populations: Primary care (PEI), mental health (ES, EMP), oncology (PEQ, PRES^2^), as a result measure in the design of specific educational interventions (DES, CES, PRES), or as a measure of empowerment of the setting (PES^1^).

Small [13] propose another tool to measure empowerment in chronic patients from a primary care perspective, based on a qualitative analysis of the concept of empowering from the view of patients with chronic diseases.

The tool is based on a model of empowerment centred on the patient that includes 5 dimensions. It contains 51 items with answers in Likert scale type of 5 points (1: completely disagree - 5: completely agree). After the psychometric analysis the tool is reduced to 3 dimensions and 47 items.

1. Positive attitude and sense of control (21 items): changes the patients experiment with regards to self-perception after diagnosis, how they reduce the impact of the disease in their lives and as a result gain greater self-control (proposal for translation of dimension: self-control or self-management).
2. Knowledge and confidence in decision making (13 items): acquire knowledge and capability to make decisions relative to disease management, to be able to participate in the process of making decisions along with the professionals and the possibility to change their preferences (proposal of translation of the dimension: shared decision making).
3. A mixture of dimensions in the 3rd factor: enabling others, knowledge and understanding and decision making, this dimension was not named (13 items).

In our country there are no validated tools which allow to measure the combination of knowledge, attitudes and abilities that enable the patient higher self-control and self-efficiency in their disease management. This lack of tools contrasts firstly, with the requests by the Health Programs from some autonomous communities [4] of searching and implementing strategies that allow empowerment in chronic patients. Secondly, the WHO has claimed the need for tools to measure the results of empowerment and for these tools to use results provided by the patients (PROMs, Patient-Reported Outcomes Measures) [6].

Consequently, due to the lack of validated tools in Spanish, the present study was designed with the objective of carrying out the transcultural adaptation and validation of a questionnaire which allows to explore the level of empowerment of patients with chronic disease. Simultaneously, it would allow to measure the effects of the interventions designed to increase the level of empowerment.

This study’s main aim is to validate the Spanish version of the questionnaire “Patient empowerment in long-term conditions” which assesses the degree of empowerment in the management of chronic diseases.

Therefore, the following will be carried out: a) transcultural adaptation of the questionnaire and b) assessment of the psychometric properties (reliability through reproducibility and internal consistency, validity of construct through convergent and divergent validity by means of comparison with other tools, questionnaire’s sensibility to change and feasibility through description of the answer from items as well as range, minimums and maximums scores obtained by the items and dimensions). The second objective of the study consists in identifying patients’ empowerment patterns with heart failure to a) identify patients’ predicting factors of basal empowerment in relation with sociodemographic, clinical and psychosocial data, b) identify predicting factors of change (improvement or deterioration) of the degree of empowerment with regards to the basal level, in relation with sociodemographic, clinical and psychosocial data, c) analyzing the relation between the level of empowerment with the incidence of hospital events, mortality and health costs.

## Methods/design

### Study design

For the first aim a quantitative, longitudinal and prospective study of pshycometric type will be carried out to validate the questionnaire. For the second aim a prospective study of cohorts will be performed.

The aim of the transcultural adaptation process of the questionnaire “Patient empowerment in long-term conditions” is that the tool is equivalent at a semantic, conceptual and content level [34, 35] to the original version.

Through semantic equivalence we wish to obtain the same meaning in each one of the items, conceptual equivalence ensures that the questionnaire measures the same theoretical construct in both cultures and the content equivalent demonstrates that each item has the same relevance in both cultures.

There are different methods to adapt the tools which include direct translation, back translation, committee assessment and pilot studies [36–38].

**Fig. 1:**
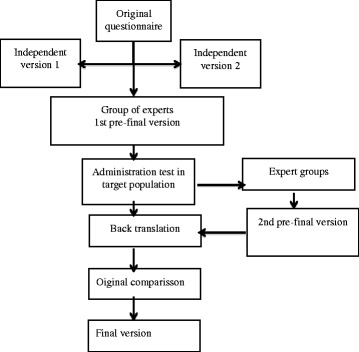
Cultural adaptation process of the questionnaire

For this procedure the EMPRO (Evaluating the Measurement ofPatient-Reported Outcomes) guidelines will be followed [39], based on the proposal of the Medical Outcomes Trust scientific committee (Fig. 1): a) contact with the author to request the use permit for the tool, b) translation of the items and the options of response in two independent versions (bilingual translators whose mother tongue is the same as the target population’s, who know the content and the aim of the questionnaire), c) conciliation and synthesis of the versions in an agreed version by the committee of experts, d) carry out the 1st pre-final version, e) administration test of the adapted pre-final version as sample of convenience (10–12 patients), f) assessment of comprehension and applicability of the adapted pre-final version, g) analysis of the results by a committee of experts and design of the 2nd pre-final version, h) inverse translation of the 2nd version to the original language by a bilingual translator, i) agreement on the back translated version with the author of the original version, j) create the final version with the author’s contribution.

### Study setting

**Fig. 2:**
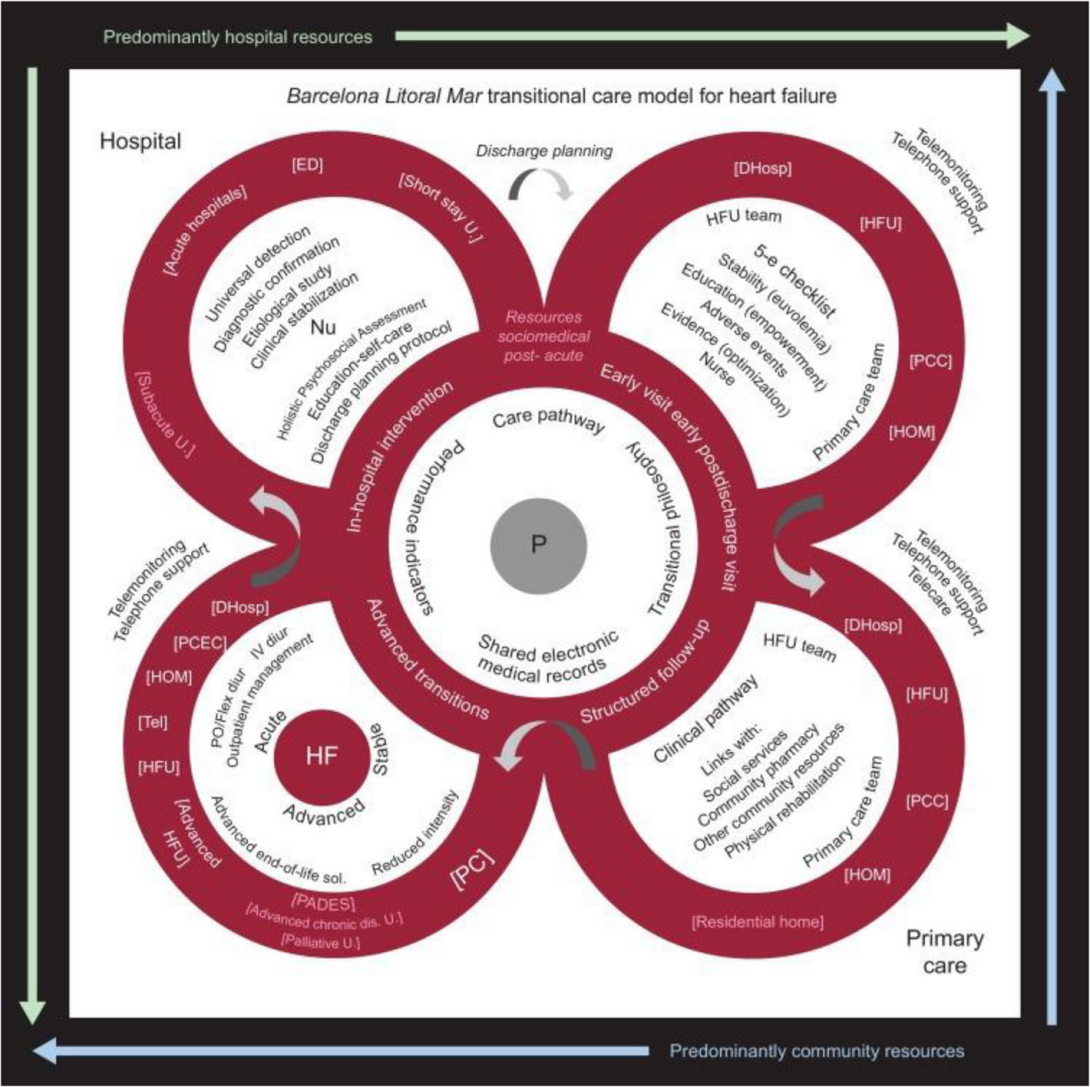
Barcelona Litoral Mar transitional care model for heart failure

A multicenter study, which will be carried out in 3 integral health areas in the urban context of Barcelona city and metropolitan area. Recruiting context is constituted by the care programs for patients with HF which exist in these health areas of the PIIC (Intervention program for an individual and collective health care in Catalonia). These programs include hospital units of Heart Failure and Primary Care of the corresponding territory, they provide a centred structured follow-up in nursery and the participation of multidisciplinary teams (Fig. 2, Table 1) [40, 41].

**Table 1:** Contents of the educational program

	Admission	Follow-up at discharge
*Educational material*	*Individual educational sessions*	*Individual sessions* (*in*-*person*, *over the phone*, *virtual*)
Guide for patients with HF	Heart failure and symptoms	Session 1 (2nd week) : Assess self-care, explain signs of alarm and flexible diet of diuretics
	Diet: salt and liquids	
Table of exercises	Weight control (meaning of sudden increase in weight)	Session 2 (4th week): reinforce information on diet habits (salt and liquids) and healthy habits
Notebook for weight control	Signs of alarm: identify signs of decompensation and prevent situations of risk	Session 3 (6th week) pharmacological treatment
Control of liquids intake	Pharmacological treatment: Effects, Doses and time of administration, Secondary effects, signs of intoxication or intolerance, Self-management of diuretics or Antihypertensive.	Session 4 (8th week): Assessment of knowledge on alarm signs, flexible diet of diuretics, diet (salt and liquids), healthy habits and medication
	Promote giving up toxic habits	
	Exercise and rest	

### Participants

All patients with confirmed diagnosis of Heart Failure (HF) according to the criteria of the European Cardiology Society [42].

Inclusion criteria: a) patients who are admitted in hospital with main diagnosis of HF at basal or redeteriorating who will carry out a follow-up at discharge in the different care areas of PIIC, b) patients who are willing to sign their informed consent.

Exclusion criteria: a) patients with acute coronary syndrome, b) patients with valvular heart disease caused by surgery to correct secondary Heart Failure in a period inferior to 3 months, c) patients who are not able to participate in the study due to the clinical situation or cognitive alterations, d) language barrier which hinders the duly completion of the questionnaire, e) patients under 18.

### Sample size

The number of individuals who must participate for the validation of the questionnaire must be between 2 and 10 times the number of items the instrument has. Hence, to carry out the pilot test 97 participants will be included [43]. Estimating the possibility of a 20% of loss, the number of individuals that need to be included in the study are 121.

To calculate the size of the sample with regards to the 2nd objective, the level of self-care of our population is taken as reference. Patients with a positive level of self-care would present an average score of 197 points (standard deviation of 80 points) in the empowerment test. While the patients with higher scores in the self-care test (which indicate poorer self-care) will present an average score of 165 points (standard deviation of 60 points). Being the proportion of 1:5 between the patients of the two groups, to demonstrate significant differences in the empowerment test between these two with an error of β = 0,20 y un error α = 0,05, 55 patients are required in the group with a positive level of self-care and 275 patients in the other, in total 330 patients with a complete assessment of the tools at basal.

For inclusion a consecutive non-probability sampling will be carried out.

### Study measures

The variables obtained from each participant will be:

- Sociodemographic information: age, gender, marital status, education, home sharing, presence of a carer.
- Clinical information: etiology of HF, functional class according to the New York Heart Association (NYHA), left ventricular ejection fraction (LVEF), severity of the symptoms, comorbidities through Charlson’s comorbidity index [44], years/months passed since diagnosis, pharmacological treatment.
- Psychosocial analysis: Hospital Anxiety and Depression Scale (HADS) [45]
- Quality of life scale: Minnesota Living With Heart Failure questionnaire (MLWHF) [46]. A self-administered Health Related Quality of Life tool, specific for HF composed of 23 items and 7 dimensions: physical limitation, symptoms (stability, frequency and severity), self-care, quality of life and social limitation. The answer options of the items are Likert type scales of 5, 6 and 7 points (1–5, 1–6 and 1–7), the score in each one of the dimensions has a theoretical range that goes from 0 to 100, 100 being the best health condition. The mentioned domains have various questions: the domain on physical limitation has 1 question broken down into 6 items. With regards to frequency, the domain that includes the symptoms comprises questions 3, 5, 7 and 9. Severity in questions 4, 6 and 8 and stability or change in time in question 2. The domain that collects the information on self-care includes questions 10 and 11, and the domain that inquires on quality of life includes questions 12–14. While the domain on social limitation includes question 15 which contains 4 items.
- Self-care level: European Heart Failure Self-care Behaviour Scale (EHFScBS) [47]. This scale consists of an administrated questionnaire with 12 items which deal with different aspects of patients’ self-care. Each item is scored from 1 (I completely agree/always) to 5 (I completely disagree/never). The global score may vary, from 12 (better self-care) to 60 (worse self-care).
- Self-efficiency level: General Self-efficiency Scale [48]. Assesses stability feeling of personal competence to manage efficiently a great variety of stressful situations. The only change made in the original questionnaire, which consists of 10 items with Likert type scales of 4 points, was the format of answer to scales of 10 points.
- Level of empowerment: Patient empowerment in long-term conditions [13]

### Procedure and data collection

**Fig. 3:**
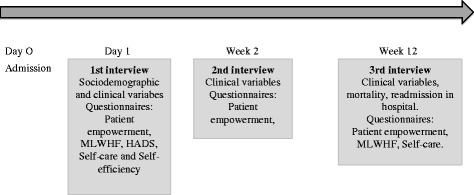
Data collection method

An initial interview and two follow-up interviews at week 2 and 12 after inclusion (Fig. 3).

Initial interview: with the coordinator nurse of HF in the hospital unit, the first working day after hospitalization and before the educational program. At this point the sociodemographic and clinical variables will be collected as well as the basal questionnaires. The questionnaires Patient empowerment, MLWHF, HADS, EHFScBS and Self-efficiency are handed out. The questionnaires will be collected between 24 and 48 h after their delivery. The data collected from the tools will allow to assess the internal consistency of the questionnaire, its validity, sensibility (pre-intervention) and feasibility.

2nd interview: carried out at 2 weeks from the initial interview. The information collection will be carried out by the nurses responsible for each care field of the program. The Patient empowerment questionnaire will be handed out and will be collected before the interview ends. The data collected will allow to analyse reproducibility (re-test).

3rd interview: carried out at 3 months from the 2nd interview. The context, people in charge, and methods of data collection will be the same as in interview 2. Clinical information will be gathered to assess, improvement or worsening of the patient. Patient empowerment, MLWHF and EHFScBS questionnaires will be handed out. The data gathered on tools will allow to assess sensibility (post-intervention).

### Data analysis

A descriptive analysis will be carried out of the features of the total sample and of the sub-samples “stability” and “improvement”. The qualitative variables will be analysed through the description of frequencies of each one of the categories whilst, the quantitative variables will be described with the average, median and typical deviation.

The psychometric analysis of the tool will be carried out by means of reliability, construct validity, sensibility towards change and feasibility.

Feasibility being the property of a questionnaire to distinguish up to what point a variable fluctuates as the result of an error in the measurement or a real change, in other words, the degree an instrument is free from aleatory error. The criteria used to measure feasibility are internal consistency and reproducibility or stability. To evaluate internal consistency Cronbach’s alfa coefficient (initial evaluation) will be used as indicator and reproducibility test-retest by means of the intraclass correlation coefficient [35, 46, 49] from the data gathered during the 1st and 2nd interviews.

The construct validity analyses the degree of correlation of the questionnaire with other tests that measure equal or similar aspects (convergent validity) or different ones (divergent validity). For this matter, a matrix of correlations of the dimensions of Patient empowerment in long-term conditions/General Self-Efficacy Scale/MLWHF/EHFScBS will be designed. Pearson’s correlation coefficient is used in means of statistics (data from the 1st interview) [35, 39, 46].

Sensibility to change is evaluated through the size of the effect that relates the average (Wilcoxon test) of the differences amongst the scores before (1st interview) and after intervention (3rd interview) [35, 46, 49].

For each questionnaire is evaluated the range of scores observed, the percentage of patients with an unanswered item in each domain (as a measure of feasibility) and the percentage of patients with either a maximum score (as a measure of the ceiling effect) or a minimum score (as a measure of the floor effect) [46, 49].

To describe changes in the sample through time the Student t-test will be used, for paired samples, the presence of unique empowerment trajectories will be explored using models of “growth mixture modelling” type (GMM) [50, 51].

Statistics meaning is established at equivalence 5% (*p* < 0,05). Data will be analysed with software Statistical Analysis System (SAS) 9.4.

### Validity and reliability

All the questionnaires used in this study have been validated in the Spanish context except for the questionnaire proposed in this study. These questionnaires will be used during a structured follow-up of the patients with HF. Therefore, nurses will be trained in their use and the three Units of HF that participate share the same procedures and clinical practise.

There will be a coordinator in each hospital who will validate the data obtained to ensure quality, and an expert coordinator who will organise the hospitals included in the study and the data collection.

## Discussion

Empowerment is a multidimensional concept that offers information on the ability of self-care and self-management of the disease but, it also gathers psychosocial aspects which are decisive in the perception of quality of life. Therefore, it is necessary to carry out the validation of the tool that measures this concept. A tool that we lack, and will offer us the results that we could only gather previously with different tools, and that often did not provide the information on all the dimensions needed.. Moreover, this tool goes hand in hand with the objective of patient centred healthcare, for which both patients and professionals must understand their new role, patients who are more active and professionals who act as facilitators of this process, to promote a relation of association, to help patients be more active by transferring knowledge and abilities. This implies the need of modifying the pillars of health education since it is not only about transferring knowledge but also of achieving a change in behaviour and developing strategies of confrontation. Therefore, professionals must incorporate in their practice communicative knowledge and abilities that allow changing the paradigm.

This project also suggests the need of creating and fomenting synergies between the different levels of health care, putting the patient in the focus rather than the health structures.

### Limitations

The main limitation of the study lies in the features of the validation process of the original tool with regards to the metric properties. Since in the original version, the internal consistency of the questionnaire and the validity of the construct were evaluated but the reproducibility and sensibility to change were not [13]. With regards to the disease, the original questionnaire was validated in a chronic heterogeneous patient population (diabetes, asthma and ischemic heart disease). Whilst in this study it has been decided to carry out validation through patients with HF, in later studies chronic patients with diverse pathologies need to be included.

Regarding the identification of the patterns and trajectories of empowerment, this study has a cohort of 3 months follow-up, taking into account that during this period the patients receive educational intervention, it would be interesting to carry out a follow-up of the cohort during a longer period of time.

On the other hand, it is an observational study that intends to quantify the associations produced between empowerment and clinical, sociodemographic and psychosocial data. Therefore, it will be necessary to design future experimental studies that provide additional information between the improvement in modifiable factors and the level of empowerment.

## Conclusion

By setting a scale that indicates the degree of empowerment, we will be able to identify those groups of patients with a low empowerment level at an early stage which could consequently, become a risk group due to poor management of the disease, with a high rate of decompensation and higher use of health resources. Will allow us to act in a preventive way, through specific educational programs which include innovative methods of communication in those patients with profiles that show a low empowerment pattern, or an unfavourable empowerment change. It will also be a tool which will allow us to measure the effects of the designed interventions to increase the level of empowerment.

## Abbreviations

CES: Client empowerment scale
DES: Diabetes empowerment scale
EHFScBS: European heart failure self-care behaviour scale
EMP: Empirical empowerment measure
EMPRO: Evaluating the measurement of patient-reported outcomes
ES: Empowerment scale
GMM: Growth mixture modelling
HADS: Hospital anxiety and depression scale
HF: Heart failure
MLWHF: Minnesota living with heart failure questionnaire
NYHA: New York heart association
PEI: Patient enablement instrument
PEQ: Psychological empowerment questionnaire
PES1: Patient empowerment scale
PES2: Patient empowerment scale
PIIC: Intervention program for an individual and collective health care in Catalonia
PRES: Pregnancy related empowerment scale
PROMs: Patient-reported outcomes measures
SAS: Statistical analysis system
WHO: World health organization
